# Cost-effectiveness of dialectical behaviour therapy vs. enhanced usual care in the treatment of adolescents with self-harm

**DOI:** 10.1186/s13034-018-0227-2

**Published:** 2018-04-30

**Authors:** Egil Haga, Eline Aas, Berit Grøholt, Anita J. Tørmoen, Lars Mehlum

**Affiliations:** 1 National Centre for Suicide Research and Prevention, University of Oslo, Sognsvannsveien 21, Bygg 12, 0372 Oslo, Norway; 2 Department of Health and Health Economics, University of Oslo, Oslo, Norway

**Keywords:** Cost-effectiveness, Self-harm, Psychotherapy, Longitudinal, Randomised trial

## Abstract

**Background:**

Studies have shown that dialectical behaviour therapy (DBT) is effective in reducing self-harm in adults and adolescents.

**Aims:**

To evaluate the cost-effectiveness of DBT for adolescents (DBT-A) compared to enhanced usual care (EUC).

**Methods:**

In a randomised study, 77 adolescents with repeated self-harm were allocated to 19 weeks of outpatient treatment, either DBT-A (*n* = 39) or EUC (*n* = 38). Cost-effective analyses, including estimation of incremental cost-effectiveness ratios, were conducted with self-harm and global functioning (CGAS) as health outcomes.

**Results:**

Using self-harm as effect outcome measure, the probability of DBT being cost-effective compared to EUC increased with increasing willingness to pay up to a ceiling of 99.5% (threshold of € 1400), while with CGAS as effect outcome measure, this ceiling was 94.9% (threshold of € 1600).

**Conclusions:**

Given the data, DBT-A had a high probability of being a cost-effective treatment.

## Background

Repeated self-harm is strongly associated with mental health problems [1, 2], and a large proportion of self-harming adolescents report having been in contact with mental health services, if not necessarily in relation to their self-harm episodes [3–5]. Psychosocial treatments that effectively reduce self-harm in adolescents have only recently emerged. Such treatments seem to be characterised by a sufficient dose of treatment and family involvement [6]. Repeated self-harm is resource-demanding, as it involves a broad range of health services for shorter or longer periods of time. Resources are, however, always limited, and there is a strong consensus that our clinical priorities should be made on the basis of the severity of the disorder, expected benefits of the treatments, and assessment of the relationship between costs and effects. Studies of cost-effectiveness involve the systematic measurement of the inputs (treatment costs) and outcomes (health) of two alternative treatments, commonly the new experimental treatment and standard treatment. The subsequent comparative analysis provides decision-makers with information on between-treatments differences with respect to costs and health effects. The results thus form the basis for evaluating whether the new treatment produces a better health effect to a lower or similar cost compared to standard treatment, alternatively that a higher cost is acceptable for added health effect. In the present study the cost-effectiveness of DBT-A is analyzed based on the incremental cost-effectiveness ratio (ICER), given by the ratio of between-group differences in costs and effects.

Several trials have shown that dialectical behaviour therapy (DBT) is effective in reducing self-harm [7–11] compared to treatment as usual (TAU). Two previous RCT studies, both comparing DBT with treatment as usual (TAU) over a period of 12 months, have included an economic evaluation. A study with female adult patients (N = 44) showed that DBT treatment incurred significantly higher psychotherapy costs, but lower inpatient care and emergency room costs than TAU over a period of 12 months. However, the results indicated no statistically significant differences in total treatment costs [12]. Another economic evaluation of DBT (age > 16 years, N = 40), yielded a similar result as it showed no significant differences in total treatment costs [13]. In a review of the cost-effectiveness of treatments for people with borderline personality disorder (BPD), treatment studies were included by estimating cost data on the basis of available resource use data thus enabling analyses of cost-effectiveness. The authors conclude that none of the reviewed treatments, including DBT, were cost-effective, but that DBT has a potential for being cost-effective [14]. A shortened version of DBT, delivered in the outpatient setting, has been adapted for adolescents (DBT-A). With a strong focus on teaching distress tolerance skills and enhancing family functioning, the treatment is expected to use more resources in the outpatient setting than usual care, one of the aims being to reduce the need for hospitalizations. Recently, we have shown that DBT-A is more effective than enhanced usual care (EUC) in reducing frequency of self-harm episodes [15, 16]. To our knowledge, no study has conducted an economic evaluation of DBT-A. It is important to establish whether such a relatively brief intervention with intensified use of resources would lead to reduced needs for resources in the longer term, and particularly whether DBT-A is associated with a reduced need for hospitalizations, thus, reducing treatment costs substantially. The aims of the present study were: to assess the total treatment costs of DBT-A compared to EUC, both over the treatment trial period of 19 weeks and over a subsequent follow-up year of 52 weeks, and to evaluate in a health care perspective the cost-effectiveness of DBT-A compared to EUC, with number of self-harm episodes and global functioning as health outcomes. To examine the economic impact of the intervention after the relatively short trial period the cost-effectiveness analysis will be conducted on the entire observational period from treatment start to follow-up assessment, altogether 71 weeks.

## Methods

Methods have been described in separate papers [15, 16]. The core issues relevant to this cost-effectiveness study are presented below. The study is registered at ClinicalTrials.gov (Identifier NTC00675129).

### Design

Participants were randomised to receive either DBT-A or EUC, stratified according to the presence of major depression, suicide intent at the most severe self-harm episode in the 4 months prior to enrollment, and gender.

### Participants

A total of 77 adolescents (39 to DBT-A and 38 to EUC) were enrolled, from June 2008 to March 2012, mainly from child and adolescent psychiatric outpatient clinics in the Oslo area. Inclusion criteria were repeated self-harm (two or more episodes, the last episode within the past 4 months), age 12–18 years, and meeting at least three criteria of borderline personality disorder (assessed by SCID-II). The study was approved by the Regional Committee for Medical Research Ethics, South-East Norway. All patients and parents provided written informed consent prior to inclusion in the study.

### Treatments

All participants received 19 weeks of treatment (trial period) in one of the publicly funded child and adolescent outpatient psychiatric clinics in the Oslo region/Norway. As is all publicly funded health care in Norway, treatments were free of charge for the participants in both treatment conditions.

The patients allocated to DBT-A received treatment according to the adolescent version of DBT [17]. The programme consisted of 19 weeks of weekly sessions (60 min) of individual therapy and weekly sessions (120 min) of skills training in a multifamily format. Family therapy sessions and telephone coaching were provided as needed according to the DBT-A protocol [18]. After 19 weeks, DBT-A treatment was ended and in cases where further treatment was needed, patients were referred to standard outpatient treatment (non-DBT) in one of the participating clinics. EUC was non-manualized, but was mainly psychodynamical or cognitive behaviour-oriented therapy, enhanced for the purpose of the trial through providing all therapists with training in suicide risk assessment and management and implementing a patient safety protocol [15]. Furthermore, EUC therapists were required to provide weekly treatment over a period of a minimum of 19 weeks. The termination of EUC-patients’ treatment was decided by each therapist, so that outpatient treatment was continued beyond 19 weeks when needed. In the follow-up period (week 20–71) the participants in both groups received standard outpatient treatment as needed, which would be of different length and frequency. Some of the patients did not receive any outpatient treatment (23% of the DBT-A patients and 14% of the EUC patients).

### Health outcomes

The participants were clinically assessed before treatment-start, at the end of the trial (19 weeks), and at a follow-up assessment 52 weeks after end of the trial, so that the entire observational period was 71 weeks. The clinical outcomes were evaluated by using the Lifetime Parasuicide Count (LPC) interview [19] for number of self-harm episodes (from treatment start to follow-up assessment), and the researcher rated Children’s Global Assessment Scale (CGAS) [20] for global functioning.

### Costs

Data on outpatient treatment resources (number of individual therapy sessions, family therapy sessions, group sessions, telephone consultations and the amount of medication) were collected from clinical records for the intervention period (week 0–19). Additionally, we monitored use of other health services due to self-harm or risk of self-harm (in the results section referred to as emergency treatment), which included inpatient treatment, emergency room visits, and general practitioner (GP) consultations. These data were collected from the adolescents on the basis of both interview and self-report, as well as from registry data obtained from the National Patient Registry (NPR). In the follow-up period (week 20–71) data on outpatient treatment and inpatient treatment were obtained from the NPR and from self-report questionnaires and interview. Data on GP consultations and emergency room visits were based on self-report and interview for this period.

The National Patient Registry (NPR) contains information on specialized treatment in psychiatric outpatient clinics and inpatient hospitalizations (psychiatric and somatic). The registry provides reliable records of resource use per patient, since the accurate registering of treatment contacts is mandatory and is the basis for funding of the clinics.

The data on the use of health service resources were collected over a 4-year period. The costs per resource unit were estimated on the basis of cost information from the financial year 2012. Costs are presented in EUR, converted from NOK by the average exchange rate of 2012. The mean total cost per patient in each group was estimated for the trial period (week 0–19) and for the follow-up period from end of trial period to follow-up assessment (week 20–71). Total treatment costs for the entire observational period from baseline to follow-up assessment (71 weeks) were calculated on the basis of these estimates. The estimation of cost for one specific resource unit, e.g. one individual therapy session in an outpatient clinic, was based on an approach that includes all actual costs that were required to produce the total number of individual therapy sessions within a given time period, divided by the number of sessions that were produced during that period. Thus, the cost for a resource unit includes wages for staff (clinical/administrative), equipment, IT, house rent, etc. Data on these costs were obtained from annual accounts from the participating clinics.

The specific costs related to DBT-A include the cost of telephone coaching (implying availability after regular working hours) and weekly therapist team consultations. The average cost per patient for telephone coaching was estimated on the basis of an annual extra fee which each therapist in the participating DBT-A teams received, and was added to the total outpatient cost (week 0–19) for each DBT-A patient. Similarly, the average cost per patient for DBT-A therapist team consultation was estimated and added to the outpatient cost (week 0–19) per DBT-A patient. Since there was no available data on supervision received by the EUC therapists, we have assumed that supervision received by EUC therapists was less resource-intensive compared to DBT-A by a factor of 0.5 (based on a previous economic evaluation of DBT [14]), and added this average cost to all EUC patients.

The average unit cost of one general practitioner (GP) visit (due to self-harm or risk of self-harm) was estimated based on information from the Norwegian Health Economics Administration (HELFO), and is the sum of what each patient pays the GP for the consultation, the amount of health insurance reimbursement the GP on average receives per consultation, and the average annual reimbursement the GP receives from the municipality per consultation. Data on the use of medication was collected for the trial period, and costs per patient were estimated on the basis of price per tablet for a specific psychotropic drug used by the patient (cf. records of Norwegian Medicines Agency [21]) and assumed number of tablets used, i.e. the patient’s days of receiving medication treatment and recommended daily dosage, as per The Norwegian Pharmaceutical Product Compendium [22].

### Statistical analyses

Analyses were carried out on an intention-to-treat basis. Means and standard deviations or median and interquartile ranges were computed for normally and non-normally distributed clinical/sociodemographic variables. Between-group differences were tested by independent samples *t* tests or Mann–Whitney *U* tests. Differences between group proportions were tested by Pearson’s Chi squared or Fisher’s exact tests.

Costs of treatment are presented as mean total treatment costs per patient. Long inpatient hospitalizations incur high costs by relatively few patients, so that the costs for a single patient may affect the mean of the treatment group substantially. Such hospitalizations have been treated as rare but plausible events, and we have presented results regarding emergency treatment costs both with and without costs incurred by hospitalizations.

For analysis of cost-effectiveness we estimated incremental cost-effect ratios (ICER). The ICER is given as the difference in mean costs ($\overline{{\text{C}}} _{{{\text{DBT}}}}  - \overline{{\text{C}}} _{{{\text{EUC}}}}$) divided by the difference in mean effect ($\overline{{\text{E}}} _{{{\text{DBT}}}}  - \overline{{\text{E}}} _{{{\text{EUC}}}}$ on a given health outcome), i.e. ${\text{ICER}} = \overline{{\text{C}}} _{{{\text{DBT}}}}  - \overline{{\text{C}}} _{{{\text{EUC}}}} /\overline{{\text{E}}} _{{{\text{DBT}}}}  - \overline{{\text{E}}} _{{{\text{EUC}}}}$. A treatment is considered cost-effective if the treatment is more effective at a lower or similar cost than the comparator. The more effective treatment may also be considered cost-effective despite a higher cost, depending on the willingness-to-pay for health gains [23].

**Fig. 1:**
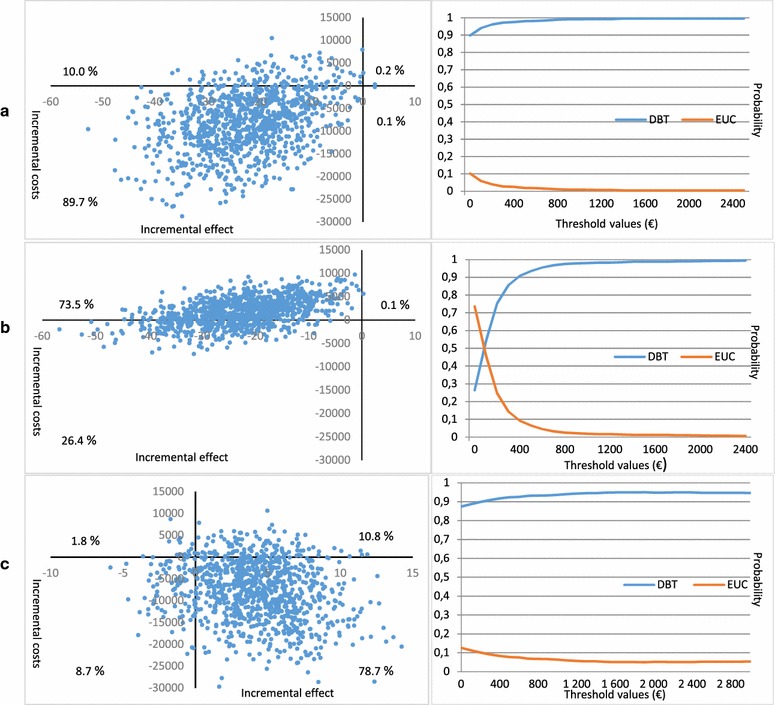
The figure shows plots of simulated ICERs, mean incremental costs on the y-axis, and mean incremental effect on the x-axis (per bootstrap sample, 1000 replications), on the left hand side. On the right hand side, the corresponding CEACs show changes in probability of DBT-A being cost-effective compared to EUC (y-axis) as a function of increasing threshold values (x-axis). **a** Plot of simulated ICERs and CEAC with incremental total treatment costs and mean incremental effect in terms of mean number of self-harm episodes. Note that increased effect is indicated by negative values on the x-axis. **b** Plot of simulated ICERs and CEAC with incremental outpatient costs (emergency treatment costs excluded) and mean incremental effect in terms of mean number of self-harm episodes. Note that increased effect is indicated by negative values on the x-axis. **c** Plot of simulated ICERs and CEAC with incremental total treatment costs and mean incremental effect in terms of change in global functioning (CGAS)

Because of the difficulties related to estimation of confidence intervals for the ICER [24], we have used bootstrapping to simulate a distribution of mean incremental costs and mean incremental effects, thus illustrating the uncertainty of the point estimate of the ICER. This was done by bootstrapping the costs and effect for each group separately (1000 replications). Incremental cost ($\Delta \overline{{\text{C}}}  = \overline{{\text{C}}} _{{{\text{DBT}}}}  - \overline{{\text{C}}} _{{{\text{EUC}}}}$) and incremental effect ($\Delta \overline{{\text{E}}}  = \overline{{\text{E}}} _{{{\text{DBT}}}}  - \overline{{\text{E}}} _{{{\text{EUC}}}}$) were calculated for each bootstrap sample and were plotted on the incremental cost-effectiveness plane (see Fig. 1), where each data-point represents one simulated $\Delta \overline{{\text{C}}}$ (y-axis) on $\Delta \overline{{\text{E}}}$ (x-axis). Finally, cost-effectiveness acceptability curves (CEAC) were constructed to summarize the uncertainty in cost-effectiveness estimates [25]. The CEAC represents the probability that DBT-A is cost-effective compared to EUC with increasing threshold values of willingness to pay for one unit incremental effect.

In the efficacy study group-differences in self-harm episodes were analysed separately for the intervention period and the follow-up period. Mixed-effect Poisson regression with robust variance was used to test for differences [16]. For estimation of incremental effectiveness in terms of self-harm, to be included in the cost-effectiveness analysis, we assumed that the groups had the same mean number of self-harm episodes at baseline, so that the effect difference was given by the difference in the mean total number of self-harm episodes per group from treatment start to the 71 weeks’ assessment.

We have missing data for some participants on specific sub-categories of outpatient treatment costs (e.g. for five patients on phone calls to patients). We also had missing data for the main cost categories: one patient in the intervention period and three patients in the follow-up period for outpatient treatment costs, and two patients in the follow-up for emergency treatment costs. We have used the mean cost for the patient’s treatment group to impute missing data. Missing data on self-harm episodes (two DBT-A patients and six EUC patients) have been imputed by using the expectation–maximization (EM) method. All analyses were performed with STATA 13 [26] and IBM SPSS Statistics 22 for Windows [27].

## Results

### Baseline characteristics

Mean age of the 77 patients was 15.6 years (*SD *= 1.5) and 88.3% were girls. There were no differences between the 39 allocated to DBT-A and the 38 participants allocated to EUC on any of the reported sociodemographic and clinical variables before treatment start (Table 1). There were also no between-group differences with respect to proportion of patients having received any psychiatric treatment (68.0% of the total sample) and having been admitted to inpatient psychiatric treatment (7.8% of the total sample) prior to participation in the study.

**Table 1:** Sample characteristics before treatment start

	DBT-A (N = 39)		EUC (N = 38)		Total sample (N = 77)	
	N	%^a^	N	%^a^	N	%^a^
Female sex	34	87.2	34	89.5	68	88.3
Child protection (current)	6	15.4	7	18.4	13	16.9
Child protection (past)	10	26.3	11	28.9	21	27.6
Past psychiatric treatment	28	73.7	23	62.2	51	68.0
Past inpatient psychiatric treatment	3	8.6	3	7.9	6	7.8
Current DSM-IV Axis I and II diagnoses						
Major depression	9	23.1	8	21.1	17	22.1
Other depressive disorder	16	41.0	13	34.2	29	37.7
Panic disorder	2	5.1	5	13.2	7	9.1
Posttraumatic stress disorder	7	17.9	6	15.8	13	16.9
Any anxiety disorder	18	46.2	15	39.5	33	42.9
Any substance use disorder	1	2.6	1	2.6	2	2.6
Any eating disorder	3	7.7	3	7.9	6	7.8
Borderline personality disorder	10	26.3	5	14.3	15	20.5
Suicide attempts, last 4 months (n)	11	28.2	9	23.7	20	26.0

	Mean	SD	Mean	SD	Mean	SD
Age (years)	15.9	1.4	15.3	1.6	15.6	1.5
C-GAS score	55.3	8.0	57.9	10.1	56.1	8.3
CBCL total, by parent (n)	69.6	11.0	68.4	8.9	69.0	9.8
Suicide attempts, lifetime (n)^c^	2.1	5.2	1.3	2.8	1.7	4.2
Non-suicidal self-harm, lifetime (n)^b^	49.5	159.5	25.0	45.5	34.0	88.0

^a^ Due to missing data in some cells there were slight variations in the percentage basis

^b^ Median and interquartile range

^c^ The median was zero for both groups. The interquartile ranges were 1.0 and 1.3 in the DBT and EUC group, respectively

### Main results of the efficacy study

In the first 19 weeks, DBT-A was superior to EUC in reducing the number of self-harm episodes and the level of suicidal ideation and depressive symptoms [15]. At 71 weeks, participants who had received DBT-A still had a statistically significantly larger reduction in self-harm episodes than participants in the EUC-group, however for the other outcomes there were no longer significant differences; this was caused by EUC participants having reached an equal level of improvement over the 1 year follow-up interval [16].

### Incremental costs

DBT-A had significantly higher outpatient treatment costs at 19 weeks (Table 2), mainly due to the costs incurred by the DBT-A multifamily skills training (group sessions). The costs of emergency treatment due to self-harm or risk of self-harm were higher in the EUC group due to one long hospitalization. Because of the low number of patients and incidents, the difference was not tested statistically. The average cost per patient for medication in the trial period was included in the outpatient treatment costs and was 7 € in both groups (SD = 42 for the DBT-A group and SD = 19 for the EUC group). DBT-A incurred higher total treatment costs. The mean difference € 2981 (95% CI = − 4666 to 10,629) was statistically significant (*p* < 0.000).

**Table 2:** Outpatient, emergency and total treatment costs (EUR) per patient, week 0–19 (trial), week 20–71 (follow-up) and week 0–71 (entire period)

	Week 0–19				Week 20–71				Week 0–71			
	DBT-A	EUC	Mean diff (SE)	p	DBT-A	EUC	Mean diff (SE)	p	DBT-A	EUC	Mean diff (SE)	p
	Mean (SD)	Mean (SD)			Mean (SD)	Mean (SD)			Mean (SD)	Mean (SD)		
Outpatient costs	15,850 (5758)	9566 (5782)	6284 (1333)	0.000^a^	5367 (7079)	9938 (9894)	− 4571 (1969)	0.010	21,217 (10,906)	19,504 (14,291)	1713 (2892)	0.555^b^
Emergency treatment costs^a^	349 (1726)	3651 (21,644)	− 3303 (3476)		541 (2996)	6757 (22,503)	− 6215 (3682)		890 (4701)	10,408 (30,440)	− 9518 (4803)	0.261
Emergency treatment costs, hospitalizations excluded	24 (73)	90 (187)	− 66 (33)		63 (171)	20 (74)	43 (30)		87 (206)	110 (217)	− 23 (46)	0.659
Emergency treatment: number of patients and incidents	6 patients (2 inpatients) 10 incidents	10 patients (2 inpatients) 23 incidents			8 patients (1 inpatient) 11 incidents	8 patients (6 inpatients) 10 incidents			11 patients (3 inpatients) 21 incidents	13 patients (8 inpatients) 33 incidents		
Total treatment costs	16,199 (6669)	13,217 (23,006)	2981 (3839)	0.000	5909 (8266)	16,695 (27,042)	− 10,787 (4582)	0.007	22,107 (13,358)	29,912 (40,179)	− 7805 (6860)	0.508

^a^ Costs of emergency treatment due to self-harm or risk of self-harm, including GP consultations, emergency department visits and hospitalizations

^b^ t test. Other tests of p values of mean difference are tested by Mann–Whitney U

The EUC patients incurred significantly higher outpatient costs than the DBT-A patients in the follow-up period (week 20–71). The EUC emergency treatment costs were higher because of one long inpatient hospitalization (difference not tested statistically). The total treatment costs were higher in the EUC group in this period, and the mean difference € − 10787 (95% CI = − 20023 to − 1550) was statistically significant (*p* = 0.007).

For the entire observation period from treatment start to follow-up assessment (week 0–71), the difference in outpatient treatment costs between the DBT-A patients and the EUC patients was not statistically significant (*p* = 0.555). Although the EUC group incurred on average higher emergency treatment costs, the difference was not statistically significant (*p* = 0.261). EUC incurred higher total treatment costs, but the mean difference € − 7805 (95% CI = − 21622 to 6012) was not statistically significant (*p* = 0.508).

### Incremental effectiveness

The EUC patients reported a mean of 22.5 (95% CI = 11.4–33.5) episodes in the 19 weeks trial period and 14.8 (95% CI = 7.3–22.3) episodes during the subsequent follow-up period, whereas the DBT-A patients reported a mean of 9.0 (95% CI = 4.7–13.2) and 5.5 (95% CI = 1.7–9.1) in the corresponding time intervals. The between-group difference was statistically significant at both time intervals (*p* < 0.05) [16]. For estimation of incremental effectiveness in terms of self-harm, we analysed the frequency of self-harm episodes for the entire observation period of 71 weeks. Since we did not have comparable data on number of self-harm episodes at baseline, the effect difference was given by the between-group difference at 71 weeks based on the assumption that baseline levels of self-harm were similar in both groups. The mean number of self-harm episodes was 15.0 (*SD* = 17.5) for the DBT-A patients and 37.5 (*SD* = 52.9) for the EUC patients. The mean effect difference was − 22.5 (95% CI = − 40.6 to − 4.3) (Table 3).

**Table 3:** Summary of costs (EURO) and effects at follow-up assessment (71 weeks)

	Total costs	Outpatient costs	Number of self-harm episodes	Change in CGAS score
	Mean (SD)	Mean (SD)	Mean (SD)	Mean (SD)
DBT-A	22,107 (13,358)	21,217 (10,906)	15.0 (17.5)	10.4 (13.4)
EUC	29,912 (40,179)	19,504 (14,291)	37.5 (52.9)	6.3 (14.9)

	Mean (CI 95%)	Mean (CI 95%)	Mean (CI 95%)	Mean (CI 95%)
Group differences	− 7805 (− 21,622 to 6012)	1713 (− 4049 to 7475)	− 22.5 (− 40.6 to − 4.3)	4.1 (− 2.3 to 10.6)
ICER, total costs/self-harm	346^a^			
ICER, outpatient costs/self-harm	− 76^a^			
ICER, total costs/CGAS	− 1904^b^			

^a^ Cost difference in EURO per reduction of one self-harm episode

^b^ Cost difference in EURO per one point improvement in CGAS score (global functioning)

Global functioning was measured by CGAS, and effect was calculated as change in CGAS from baseline to follow-up assessment (week 0–71). Mean improvement in CGAS was 10.4 (*SD* = 13.4) for the DBT group and 6.3 (*SD* = 14.9); the mean effect difference 4.1 (95% CI = − 2.3 to 10.6) was not statistically significant (*p* = 0.204).

### Incremental cost-effectiveness with number of self-harm episodes as effect outcome measure

The incremental cost-effectiveness ratio (ICER) was estimated to € − 7805/− 22.5 = € 346; i.e. the cost reduction for DBT-A compared to EUC was € 346 per reduction of 1 self-harm episode (Table 3). Bootstrapping (1000 replications) was performed, and the incremental mean cost and effect of each bootstrap sample were plotted on the incremental cost-effectiveness plane (Fig. 1a). A proportion of 89.7% of the simulated ICERs falls into the quadrant where a reduction in self-harm is achieved by DBT-A for less cost compared to EUC. Additionally, 10.0% of the simulated ICERs fall into the quadrant with better effect to a higher cost.

The cost effectiveness acceptability curve (CEAC, Fig. 1a) shows the probability of DBT-A being cost-effective in reducing the number of self-harm episodes, compared to EUC, as a function of increasing threshold values of willingness to pay for reduction of one self-harm episode. With a zero threshold, i.e. no willingness to pay, the probability of DBT-A being cost-effective is 89.8% (the proportion of the simulations below the x-axis). With increasing threshold values, the probability of DBT being cost-effective increases, since a proportion of the simulated ICERs in the quadrant above the x-axis is added to the proportion considered cost-effective. The probability of DBT-A being cost-effective increases to 97.5% with a threshold value of € 400, and up to a ceiling probability at approx. 99.5%, at a threshold of € 1400.

As noted above two long inpatient admissions in the EUC group substantially affected the total mean costs of the EUC patients. In order to study the impact of such costs on the ICER we excluded the inpatient and other emergency treatment costs for both groups, thus including only outpatient costs. The ICER was estimated to € 1713/− 22.5 = € − 76, i.e. the extra cost for a reduction of one self-harm episode was € 76. The CEAC (Fig. 1b) showed that, with costs associated with inpatient and other emergency treatments excluded from the analysis, EUC had a higher probability of being cost-effective with no willingness to pay for extra effect. With willingness to pay approximately € 100 per reduction of one self-harm episode, the probability of being cost-effective was equal for the treatment groups; with willingness to pay more than € 100, DBT-A had a higher probability of being cost-effective, i.e. a probability up to a ceiling ratio of 99.9%.

### Incremental cost-effectiveness with global functioning (CGAS) as effect outcome measure

With CGAS as effect outcome measure, the ICER was estimated to € − 7805/4.1 = € − 1904, i.e. the cost reduction for DBT-A vs. EUC was € 1904 per one point improvement in CGAS. The plot of mean costs and effects showed that a majority of the simulated ICERs falls into the quadrant with more effect at a lower cost (78.7%). The CEAC showed that the probability of DBT-A being cost-effective increases up to a ceiling of 94.9% at a threshold of € 1600.

## Discussion

This study showed that there were no statistically significant differences between DBT-A and EUC with respect to total treatment costs when taking both the treatment trial period of 19 weeks and the 1-year follow-up interval under consideration. When cost data were analysed together with our previously published outcome data showing that DBT-A was superior to EUC in reducing self-harm over the relevant time interval [16], we found that DBT-A had a probability of being cost-effective increasing from 89.8% with no willingness to pay extra for extra health gains, up to a ceiling probability at approximately 99.5% with increasing willingness to pay up to a threshold of € 1400. Thus, given the data, DBT-A had a high probability of being cost-effective compared to EUC.

DBT-A had higher outpatient treatment costs during the 19 weeks trial period, whereas EUC had higher outpatient costs during the follow-up period. It is an important finding that the intensified use of resources during the intervention period was followed by a subsequent reduced need for treatment in the follow-up period for the DBT-A group. Our efficacy study showed that DBT-A resulted in a more rapid improvement during the intervention period [15]. The finding that DBT-A improved health at 19 weeks and that there were no statistically significant between-group differences in outpatient treatment costs at 71 weeks, suggests that the initial extra use of resources in the DBT-A group gave good value for money.

Two previous studies have shown higher outpatient psychotherapy costs for DBT compared to the control group [12, 13]. In these studies, the original DBT program for adults was used, so that the intervention had longer duration. The patients in these studies were mainly adults, and although comparison between adult and adolescent samples should be done with caution, our findings suggest that a shorter DBT intervention period may be favourable both from an effect- and a cost-perspective.

There were no between-group differences with respect to emergency intervention costs other than hospitalization. The EUC group incurred considerably higher costs for hospitalizations due to two long inpatient stays. When including the costs of hospitalization, the total treatment costs were higher for EUC for the entire period, but the difference did not reach statistical significance, partly because the impact of long hospitalization costs are ruled out in the rank-order test (Mann–Whitney *U*). DBT-A has a specific focus on reducing hospitalization, and some previous studies have pointed in the direction that DBT may reduce the need for such hospitalization compared to the control treatment, [7, 8, 28] although this finding has not been replicated by other studies [9, 29–31]. The observed differences in our study are difficult to interpret with respect to between-group differences due to the limited number of hospitalizations. It would not be possible to conclude whether the higher incurred costs in the EUC group were due to treatment differences or a result of mere chance.

The relatively small proportion of patients receiving inpatient treatment in the present study contrasts what has been observed in studies with adult patients [7, 8, 29, 31]. It is, however, important to note that psychiatric hospitalisation of adolescents has in general a much higher threshold in most countries, since such measures are regarded as very drastic in this age group, since most adolescents have a base for care in their own family, and clinicians will normally seek to deliver crisis interventions in an outpatient fashion. To be able to observe analysable differences, a study with more patients and/or longer duration of follow-up would be required.

In this study treatment was free of charge for the participants. Provided that the way treatment is funded in other health care systems does not lead to substantial between-group differences in total treatment costs, we suggest that the findings of the study would generalize to systems where treatment is not publicly funded. However, we may assume that certain factors could affect the costs of DBT-A compared to standard treatment, e.g. the extent to which frequency of inpatient admissions differ across systems, whether one of the treatment methods is more or less available dependent of the healthcare setting, and/or the possibility that the ratio of cost per resource unit for outpatient treatment and inpatient treatment differ across systems. It is beyond the scope of this article to fully examine this complex issue of generalizability.

### Limitations

Our sample was of limited size for a cost-effectiveness study. A larger sample combined with a longer observation period would have provided a stronger basis for detecting possible group differences, on both clinical and cost variables. Most importantly it would facilitate analysis of the use of crisis services, as mentioned above, which is a highly relevant issue for this patient group. Furthermore, we have limited the cost analyses to direct treatment costs and not included societal costs. It would be expected that productivity losses due to, e.g. parents’ extra care for their adolescents, would result in indirect costs for this patient group. The adolescents’ absenteeism from school would also be a relevant indirect cost unit to study. Although difficult to value within a limited time perspective, this would be relevant to follow-up into adulthood since non-completed education may have an impact on the ability to maintain employment, with substantial indirect costs to society. Finally, quality adjusted life years (QALYs) are commonly included in cost-effectiveness studies as a generic measure of health outcome; but this was not used in the present study, since it was not initially planned as a cost-effectiveness study. Instead we chose CGAS as a measure of global health effect.

### Strengths

The liberal inclusion criteria and the delivering of treatments in a community mental health setting with patients recruited from a defined catchment area strengthen the external validity of the findings.

The validity of the findings is increased by the randomised trial design and the rigorous procedures for data collection, providing high-quality data for health outcome measures. A further strength is the high participation rate with only two participants (one in each treatment condition) lost to follow-up at 71 weeks. Finally, the calculation of costs is based on detailed and reliable data for the most resource-intensive treatment categories (outpatient treatment and inpatient hospitalizations), directly derived from records of the clinics where the patients received treatment, as well as from the Norwegian Patient Registry. The data regarding costs for GP consultations and emergency room visits due to self-harm or risk of self-harm were based on self-report and interview, and may be less accurate because of recall bias. However, collecting the data from different sources made it possible to cross-check information, thus minimizing the effect on data quality.

## Conclusions

The findings that DBT-A had a higher probability of being cost-effective compared to EUC, and that DBT-A was superior in reducing self-harm at a similar cost, support the choice of DBT-A as a treatment for adolescents with repeated self-harm. The limited sample size and low number of inpatient admissions in our study sample call for further studies to evaluate the cost-effectiveness of DBT-A.

## Abbreviations

BPD: borderline personality disorder
CEAC: cost-effectiveness acceptability curve
CI: confidence interval
CGAS: Children’s Global Assessment Scale
DBT-A: dialectical behaviour therapy for adolescents
EM: expectation–maximization method
EUC: enhanced usual care
GP: general practitioner
ICER: incremental cost-effectiveness ratio
LPC: Lifetime Parasuicide Count
SCID-II: Structural Clinical Interview for DSM Disorders
TAU: treatment as usual

## References

[1] Nock M (2010). Self-injury. Annu Rev Clin Psychol.

[2] Jacobson C, Muehlenkamp J, Miller A, Blake Turner J (2008). Psychiatric impariment among adolescents engaging in different types of deliberate self-harm. J Clin Child Adolesc Psychol.

[3] Nock M, Green J, Hwang I, McLaughlin K, Sampson N, Zaslavsky A, Kessler R (2013). Prevalence, correlates, and treatment of lifetime suicidal behavior among adolescents. JAMA Psychiatry.

[4] Nixon M, Cloutier P, Jansson S (2008). Nonsuicidal self-harm in youth: a population-based survey. CMAJ.

[5] Tørmoen A, Rossow I, Mork E, Mehlum L (2014). Contact with child and adolescent psychiatric services among self-harming and suicidal adolescents in the general population: a cross sectional study. Child Adolesc Psychiatry Ment Health.

[6] Brent D, McMakin D, Kennard B, Goldstein T, Mayes T, Douaihy A (2013). Protecting adolescents from self-harm: a critical review of intervention studies. J Am Acad Child Adolesc Psychiatry.

[7] Linehan M, Armstrong H, Suarez A, Allmon D, Heard H (1991). Cognitive-behavioral treatment of chronically parasuicidal borderline patients. Arch Gen Psychiatry.

[8] Linehan M, Comtois K, Murray A, Brown M, Gallop R, Heard H, Korslund K, Tutek D, Reynolds S, Lindenboim N (2006). Two-year randomized controlled trial and follow-up of dialectical behavior therapy vs therapy by experts for suicidal behaviors and borderline personality disorder. Arch Gen Psychiatry.

[9] Koons CR, Robins CJ, Tweed JL, Lynch TR, Gonzalez AM, Morse HQ, Bishop GK, Butterfield MI, Bastian LA (2001). Efficacy of dialectical behavior therapy in women veterans with borderline personality disorder. Behav Ther.

[10] Verheul R, van den Bosch LMC, Koeter MWJ, de Ridder MAJ, van den Brink W (2003). Dialectical behaviour therapy for women with borderline personality disorder—12-month, randomised clinical trial in The Netherlands. Br J Psychiatry.

[11] Turner RM (2000). Naturalistic evaluation of dialectical behavior therapy-oriented treatment for borderline personality disorder. Cogn Behav Pract.

[12] Heard HL (2000). Cost-effectiveness of dialectical behavior therapy in the treatment of borderline personality disorder.

[13] Priebe S, Bhatti N, Barnicot K, Bremner S, Gagli A, Katsakou C, Molosankwe I, McCrone P, Zinkler M (2012). Effectiveness and cost-effectiveness of dialectical behaviour therapy for self-harming patients with personality disorder: a pragmatic randomised controlled trial. Psychother Psychosom.

[14] Brazier J, Tumur I, Holmes M, Ferriter M, Parry G, Dent-Brown K, Paisley S. Psychological therapies including dialectical behaviour therapy for borderline personality disorder: a systematic review and preliminary economic evaluation. Health Technol Assess. 2006;10(35). 10.3310/hta10350 16959171

[15] Mehlum L, Tørmoen A, Ramberg M, Haga E, Diep L, Laberg S, Larsson B, Stanley B, Miller A, Sund A, Grøholt B (2014). Dialectical behavior therapy for adolescents with recent and repeated suicidal and self-harming behavior and borderline traits—a randomized trial. J Am Acad Child Adolesc Psychiatry.

[16] Mehlum L, Ramberg M, Tormoen AJ, Haga E, Diep LM, Stanley BH, Miller AL, Sund AM, Groholt B (2016). Dialectical behavior therapy compared with enhanced usual care for adolescents with repeated suicidal and self-harming behavior: outcomes over a one-year follow-up. J Am Acad Child Adolesc Psychiatry.

[17] Rathus J, Miller A (2002). Dialectical behavior therapy adapted for suicidal adolescents. Suicide Life Threat Behav.

[18] Miller AL, Rathus JH, Linehan MM (2007). Dialectical behavior therapy with suicidal adolescents.

[19] Linehan M, Comtois K (1996). Lifetime parasuicide count.

[20] Shaffer D, Gould M, Brasic J, Anmbrosini P, Fisher P, Bird H (1983). A children’s global assessment scale (CGAS). Arch Gen Psychiatry.

[21] Statens legemiddelverk: Price- and reimbursement list. 2013. https://legemiddelverket.no/english/price-and-reimbursement/maximum-price#list-of-products-with-maximum-prices. Accessed 1 Sept 2013.

[22] Felleskatalogen. Felleskatalogen farmasøytiske preparater markedsført i Norge. 2015. http://www.felleskatalogen.no. Accessed 1 June 2015.

[23] O’Brien BJ, Drummond MF, Labelle RJ, Willan A (1994). In search of power and significance: issues in the design and analysis of stochastic cost-effectiveness studies in health care. Med Care.

[24] Van Hout BA, Al MJ, Gordon GS, Rutten FFH (1994). Costs, effects and c/e ratios alongside a clinical trial. Health Econ.

[25] Fenwick E, Marshall D, Levy A, Nichol G (2006). Using and interpreting cost-effectiveness acceptability curves: an example using data from a trial of management strategies for atrial fibrillation. BMC Health Serv Res.

[26] Stata Corporation (2013). STATA Statistical Software.

[27] IBM Corporation (2013). IBM SPSS Statistics for Windows.

[28] Linehan M (1993). Cognitive-behavioral treatment of borderline personality disorder.

[29] Carter GL, Wilcox CH, Lewin TJ, Conrad AM, Bendit N (2010). Hunter DBT project: randomized controlled trial of dialectical behaviour therapy in women with borderline personality disorder. Aust N Z J Psychiatry.

[30] McMain SF, Guimond T, Streiner DL, Cardish RJ, Links PS (2012). Dialectical behavior therapy compared with general psychiatric management for borderline personality disorder: clinical outcomes and functioning over a 2-year follow-up. Am J Psychiatry.

[31] McMain SF, Links PS, Gnam WH, Guimond T, Cardish RJ, Korman L, Streiner DL (2009). A randomized trial of dialectical behavior therapy versus general psychiatric management for borderline personality disorder. Am J Psychiatry.

